# Harnessing Radiation Biology to Augment Immunotherapy for Glioblastoma

**DOI:** 10.3389/fonc.2018.00656

**Published:** 2019-02-22

**Authors:** Karishma R. Rajani, Lucas P. Carlstrom, Ian F. Parney, Aaron J. Johnson, Arthur E. Warrington, Terry C. Burns

**Affiliations:** ^1^Department of Neurologic Surgery, Mayo Clinic, Rochester, MN, United States; ^2^Department of Immunology, Mayo Clinic, Rochester, MN, United States

**Keywords:** radiation, glioblastoma, GBM, PD-1/PD-L1, CTLA-4, immunotherapies, innate and adaptive immune responses

## Abstract

Glioblastoma is the most common adult primary brain tumor and carries a dismal prognosis. Radiation is a standard first-line therapy, typically deployed following maximal safe surgical debulking, when possible, in combination with cytotoxic chemotherapy. For other systemic cancers, standard of care is being transformed by immunotherapies, including checkpoint-blocking antibodies targeting CTLA-4 and PD-1/PD-L1, with potential for long-term remission. Ongoing studies are evaluating the role of immunotherapies for GBM. Despite dramatic responses in some cases, randomized trials to date have not met primary outcomes. Challenges have been attributed in part to the immunologically “cold” nature of glioblastoma relative to other malignancies successfully treated with immunotherapy. Radiation may serve as a mechanism to improve tumor immunogenicity. In this review, we critically evaluate current evidence regarding radiation as a synergistic facilitator of immunotherapies through modulation of both the innate and adaptive immune milieu. Although current preclinical data encourage efforts to harness synergistic biology between radiation and immunotherapy, several practical and scientific challenges remain. Moreover, insights from radiation biology may unveil additional novel opportunities to help mobilize immunity against GBM.

## Introduction

Glioblastoma (GBM) is a deadly and highly infiltrative tumor. It is the most common primary brain tumor in adults, causing about 3–4% of all cancer-related deaths (1). Surgery followed by fractionated radiotherapy (RT) and temozolomide (TMZ) has been standard of care for newly diagnosed GBM since 2005 (2). To date, scientific advances in genomics and immunotherapy have failed to translate into effective therapies for GBM, with median survival of just over a year from diagnosis. Once recurrence has occurred, prognosis is extremely guarded with a minority of patients responding meaningfully to second-line therapies or surviving >6 months from time of recurrence (3). Novel approaches to treat GBM are urgently needed and much effort has sought to determine whether immunotherapy may provide a useful adjunct.

Immunotherapies, epitomized by successful trials with checkpoint blockade, have been widely hailed as a breakthrough in cancer therapy over the past decade. Seminal work from the Allison laboratory in 1996 showed that the antibody-blocking cytotoxic T-lymphocyte antigen-4 (CTLA-4) could elicit regression of murine colon carcinoma and fibrosarcoma (4). Since then, several other preclinical models have further validated the effectiveness of blocking CTLA-4 and supported the clinical development of anti-CTLA-4 therapy. The first human phase III study of anti-human CTLA-4 (Ipilimumab) demonstrated improved survival in patients with advanced melanoma (5). Subsequent successes followed with antibodies against programmed cell death-1 (PD-1) and programmed death ligand-1 (PD-L1) (6, 7), confirming the broad utility of blocking inhibitory pathways that interfere with anti-tumor T cell responses.

There is a strong correlation between high somatic mutation burden and the clinical response to immune checkpoint monotherapies (8). Non-synonymous somatic mutations lead to an altered amino acid sequence and give rise to neoepitopes that can serve as neoantigens recognized by the immune system (9, 10), triggering an anticancer immune response. In contrast, GBM has a relatively low burden of neoantigens (11), yielding “cold tumors” for which clinical response immune checkpoint monotherapy is infrequently observed. The “cold” phenotype of GBM is also attributed to recruitment of immunosuppressive immune cell types and secretion of immune suppressive cytokines (12–14). Much work has sought to convert the “cold” GBM phenotype into a “hot” phenotype more responsive to immune checkpoint blockade. To this end, radiation and radiation-induced immune processes have demonstrated particular promise.

Immune infiltration is a doubled sword. Despite the benefit of immune infiltrate for a successful immune therapy response, more aggressive tumors, such as mesenchymal subtype GBM, are typically heavily infiltrated by immune cells (15). In this setting, immune cells are believed to be reprogrammed by the tumor to perform pro-tumorigenic functions. However, whether the presence of robust immune infiltrate is a cause or effect of GBM aggressiveness has been controversial. Mutations in the gene isocitrate dehydrogenase (IDH) are very common in World Health Organization classification of Grade II and III gliomas and in 10% of GBM that have evolved from lower-grade tumors (16, 17). Overproduction of oncometabolite 2-hydroxyglutarate (2HD) in the D-enantiomer is a major hallmark of these glioma subtypes (18). IDH mutation status is an important classifier in stratifying glial tumors. Patients with IDH-mutant gliomas have a substantial survival benefit following chemotherapy and radiation compared to patients with IDH wild type tumors (19). A study by Amankulor et al. used this model to help shed light on the role of immune cells in tumor aggressiveness (20). It is known that IDH-mutant gliomas have fewer tumor-infiltrating immune cells, including T cells, microglia, and macrophages, compared to IDH wild-type tumors; thus IDH-mutant tumors typically exemplify “cold tumors” and may not respond to immunotherapies. The authors generated genetically engineered mice that were identical, except for the presence or absence of IDH mutation, with concomitant increase in 2-HG levels. Decreased leukocyte chemotaxis and prolonged survival was seen in the IDH-mutant tumors supporting the concept of immune infiltration as causatively pathologic in more aggressive gliomas. Whether IDH-mutant gliomas or tumors with inherently lower immune infiltration (e.g., proneural) are inherently less responsive to immunotherapy due to their “cold” phenotype is hypothesized, but remains to be demonstrated clinically. Nevertheless, since radiation is currently standard of care for all subtypes of infiltrative glioma, potential synergy between immunotherapy and radiation is an opportunity to be exploited therapeutically. In such work, the goal will be to promote and maintain an anti-tumorigenic rather than pro-tumorigenic phenotype of recruited leukocytes, even long after completion of radiotherapy.

Preclinical data have provided robust proof of principle that radiation can boost both the local and systemic antitumor immune response to augment tumor control even at sites distant from radiation—eliciting the so-called “abscopal effect.” Although radiation and immunotherapy are both currently employed in early clinical trials of immunotherapy, it is less certain whether their potentially synergistic biology is optimally harnessed using current protocols. Emerging preclinical data suggest that established standards of care for GBM—including radiotherapy fractionation regimens, use of systemically immunosuppressing TMZ, and frequent use of steroids—may need to be revisited before the potential of immunotherapy is fully realized for GBM. This review begins by addressing the current understanding of immune-modulatory effects of radiation and highlights the salient features of the highly immunosuppressive microenvironment of GBM. We then discuss preclinical data supporting the synergistic combination of radiotherapy with immunotherapies targeting both innate and adaptive immune modulators and explore important challenges yet to be overcome in search of a clinically optimal regimen.

## GBM and The Adaptive Immune System

### Brain: No Longer an Immune-Privileged Organ

The central nervous system (CNS) has long been considered immune privileged due in part to the presence of the blood brain barrier, a unique structural feature that restricts entry of molecules and immune cells into the brain. This view was further supported by relatively low numbers of antigen presenting cells (APCs) and T cells in the brain parenchyma, as well as the historically perceived lack of lymphatic vessels to drain APC and antigen to regional lymph nodes (21). Findings in recent years have challenged long-standing thinking by demonstrating that even the healthy brain is in fact under constant immune surveillance. Brain-derived antigens can entrain peripherally-derived immune cells that in turn penetrate the blood brain barrier (22, 23). Identification of a novel CNS glymphatic system, wherein most APCs could travel from the brain into the cervical lymph nodes and prime T lymphocytes (24, 25), forced reconsideration of the supposedly immune privileged status of the CNS. The revised model is in line with empiric findings of tumor-infiltrating lymphocytes detected in human GBM after vaccination with autologous tumor lysate-pulsed dendritic cells (DCs) (26, 27). It is within this dynamic scientific era that insights are sought from the brain and tumor microenvironments to optimally harness immunotherapy for GBM.

### Immune-Suppressive Microenvironment of GBM

Tumors subvert systemic and local immune mechanisms to establish an immune tolerant microenvironment permissive to infiltration and proliferation. The following sections outline several of the immunosuppressive mechanisms defined to date; the extent to which radiation may help attenuate the immunosuppressive microenvironment of GBM is discussed in section *Radiation and GBM*.

Like many tumors, GBM express relatively low levels of histocompatibility complex (MHC) class I and II molecules, thereby minimizing display of tumor-associated antigens (28). GBM also secrete immunosuppressive cytokines, such as IL-10 and TGF-β (29). TGF-β is a pleiotropic cytokine that blocks the cytotoxic T cell response and promotes the activity of CD4+ regulatory T cells (Tregs).

Tregs express CD25+ and the transcription factor FoxP3+ (30) and may derive from the periphery (pTregs) from conventional T cells or from the thymus (tTregs) (31). Tregs can be recruited to the tumor or generated via proliferation of pre-existing Tregs in the tumor microenvironment and *de novo* conversion of tumor-infiltrating CD4+ lymphocytes (TIL) into pTregs (32, 33). Tregs exert their suppressive activity through cell surface molecules such as CTLA-4, perforin, and CD73. These inhibit maturation of APCs and block B7-CD28 co-stimulatory signals. ATP released from dying cells is pro-immunogenic, but is degraded by Tregs. In addition, Tregs can also mediate their suppressive activity via contact-independent mechanisms, secreting inhibitory cytokines that suppress effector T cell function (34).

The enzyme indoleamine 2,3 dioxygenase (IDO) can be produced by both tumor and tumor APCs, including DCs and macrophages (35), to induce immune suppression. IDO contributes to immune tolerance by catabolizing tryptophan to catabolites, such as kynurenine (36). Deprivation of the critical amino acid tryptophan and exposure to metabolites inhibits the proliferation of cytotoxic CD4+ and CD8+ T cells (37), as well as natural killer (NK) cells (38). Preclinical work by Wainwright et al. has demonstrated that GBM tumor-derived IDO increased the recruitment of Tregs and decreased survival of mice with intra-cranial tumors (39). Of note, IDO expression levels tends to positively correlate with glioma grade (40).

Although GBM is confined to the brain, patients with GBM may be profoundly immunosuppressed systemically with decreased numbers (41) and function (42) of circulating lymphocytes. GBM accumulate robust numbers of intra-tumoral activated Tregs that impede the proliferation of, and cytokine secretion by, autologous lymphocytes (43, 44). Furthermore, depletion of Tregs using anti-CD25 antibodies augmented anti-tumor CD4+ and CD8+ T cell responses (45, 46). These studies emphasize the role of GBM-associated Tregs in maintaining a systemic tolerogenic environment that impedes anti-tumor immunity.

### T Cell Exhaustion in GBM

Viruses have evolved highly effective strategies for establishing chronic infection and avoiding clearance by the immune response (47, 48). During chronic viral infections, persistent antigen exposure drives CD8+ T cells to increase the expression of inhibitory receptors, dampening their ability to clear the infection (49). This state of decreased proliferation and decreased effector function, including reduced cytokine secretion accompanied by metabolic and transcriptional changes, has been termed “exhaustion” and is also induced by cancers to avoid immune clearance (50, 51). Targeting such T cell exhaustion may be more complex in cancer due to intra-tumoral heterogeneity, resulting from stochastic tumor evolution and spatial gradients within the tumor microenvironment (51). The exhausted T cell phenotype is characterized by upregulation of multiple inhibitory immune checkpoint receptors, such as PD-1 (52), CTLA-4 (4), T cell immunoglobulin 3 (TIM-3) (53), lymphocyte-activation gene 3 (LAG-3), T cell immunoreceptor with immunoglobulin and ITIM domains (TIGIT), V-domain Ig Suppressor of T cell Activation (VISTA), and CD39 (54–56). These molecules are prominently expressed on CD8+ TILs from human GBM (57) with stably elevated checkpoint expression restricted TCR repertoire clonality throughout the stages of GBM progression (58). Under normal homeostasis, these molecules play critical immune regulatory roles in mediating tolerance to self-antigens and preventing auto-immunity (59, 60). While it has been known that multiple tumors induce T cell exhaustion to promote survival (61), the degree of T cell exhaustion in patients with GBM was recently determined to be particularly severe (57). To date, the predominant strategy investigated to attenuate T cell exhaustion has included one or more immune checkpoint inhibitors (62). However, modulating metabolic and stromal components in the tumor microenvironment may prove synergistic (51). The potential role of radiation to facilitate such modulation is discussed below.

### Role of Immune Checkpoints in GBM

Numerous preclinical studies have demonstrated efficacy of antibodies targeting CTLA-4 or the PD-1/PD-L1 axis (4, 63, 64). Subsequently, these antibodies have also demonstrated clinical benefit in multiple tumor types, particularly including “hot” tumors with innately high immunogenicity. Monotherapy with ipilimumab, an anti-CTLA-4 antibody, yielded a durable response in ~10% of patients with advanced metastatic melanoma (5). Additionally, lambrolizumab (anti-PD-1) yielded a robust and durable response in about 35% of patients with advanced melanoma (65). Based on numerous such encouraging trials, several immune checkpoint inhibitors have now been FDA approved for multiple cancers. Examples include inhibitors targeting CTLA-4 (ipilimumab), PD-1 (pembrolizumab and nivolumab), and PD-L1 (atezolizumab and avelumab), that have collectively yielded profound impacts on the management of multiple systemic malignancies.

The dysregulation of immune-checkpoint pathways in GBM has provided ample proof of principle suggesting checkpoint inhibitors could also offer a therapeutic avenue for GBM (66). Indeed, in addition to upregulation of inhibitory checkpoint molecules, such as PD-L1 on T-regs and exhausted T-cells, these are also expressed on tumor-associated macrophages and microglia (TAMs) isolated from human GBM (67). Moreover, immunosuppressive cytokines in the GBM microenvironment, including IL-10, promote expression of checkpoint inhibitor expression on GBM itself (67). Despite promising responses in a subset of patients (68), benefits of checkpoint inhibition have yet to be observed in any phase III clinical trial for GBM.

Nevertheless, immune checkpoint dysregulation alone in GBM may be insufficient to portend reliable responses via checkpoint blockade. Increasing data suggest that an elevated tumor mutational burden (69, 70) and a robust lymphocytic infiltrate within the tumor microenvironment (“hot tumors”) correlate with improved clinical response to checkpoint blockade (69, 71, 72). Indeed, consistent with the relatively immunologically “cold” nature of GBM, including modest levels of tumor neoantigens and lymphocytic infiltrate, several late stage clinical trials have failed to demonstrate clinical benefit (see Supplementary Table 1). Nevertheless, promising responses in a subset of patients continue to foster enthusiasm for harnessing checkpoint inhibitors in GBM. The portfolio of checkpoint inhibitors is continuing to expand with preclinical and efficacy data in targeting LAG-3 (73), TIM-3 (74), and TIGIT (75), each showing particular promise in combination with PD-1 inhibition. Moreover, harnessing the use of immunostimulatory strategies, such as radiation, to augment checkpoint responses has generated particularly promising preclinical data (76). The following sections offer additional details regarding the more thoroughly studied checkpoint molecules CTLA-4 and PD1/PDL1 that have provided a foundation for GBM immunotherapy efforts to date.

### Cytotoxic T-Lymphocyte Antigen-4 (CTLA-4)

T cells are typically activated when an MHC-bearing APC presents an antigenic peptide and engages a T cell receptor (TCR). Full activation of T cells requires engagement of the co-stimulatory T cell receptor, CD28, with its ligands, CD80 and CD86, expressed on APC (77). CTLA-4 primarily regulates the early stages of T cell activation. CTLA-4 begins as an intracellular protein, but upon T cell activation translocates to the immunological synapse and co-localizes with TCRs (78, 79). CTLA-4 outcompetes the co-stimulatory TCR CD28 by binding with higher affinity to the ligands CD80 and CD86 expressed on APCs (80). CTLA-4 can also limit conjugation times between T cells and APCs, limit T cell proliferation, and reduce cytokine production (81). CTLA-4 inhibits Akt phosphorylation by activating protein serine/threonine phosphatase PP2A, but does not alter phosphatidylinositol3-kinase (PI3K) activity (62, 82). The intracellular domain of CTLA-4 can recruit the protein phosphatase 2 A to decrease phosphorylation of proteins in the TCR signaling cascade (83). CTLA-4 plays a key role in maintaining immune-regulated homeostasis by enhancing suppressive functions of Tregs (84) and impeding the function of CD4+ helper T cells (85). Anti-CTLA-4 antibodies can mitigate T cell exhaustion by attenuating the inhibitory functions of CTLA-4 and suppressive actions of Tregs. Ipilimumab and tremelimumab were the first anti-CTLA-4 antibodies to enter clinical trials in patients with advanced cancer. Ipilimumab is currently FDA approved for metastatic melanoma and renal cell carcinoma.

### Programmed Cell Death-1 (PD-1) and Programmed Death Ligand-1 (PD-L1)

In contrast to CTLA-4, which largely regulates T cell activation, PD-1 plays a prominent role in inhibiting proliferation and functions of effector T cell responses. PD-1 is absent on resting naïve and memory T cells, but expressed on tumor infiltrating lymphocytes (TILs) (86). PD-1 is upregulated on activated T cells upon TCR engagement and mediates T cell suppression (87) upon binding PD-L1 (52) or PD-L2 (88). PD-L1, also known as CD274 and B7-H1, is largely undetectable in most normal tissues, but is expressed on macrophages and APCs, particularly in the context of classical (M1) activation (89). PD-L1 is elevated in tumors—not only on APCs, but also tumor cells themselves, promoting tumor cell survival (90, 91). PD-L2 expression is limited to certain immune cell types, mostly DCs, mast cells, and macrophages (87). Both PD-1 and PD-L1 are expressed on Tregs (92). Binding of PD-1 on activated T cells to PD-L1 decreases TCR-mediated signaling by antagonizing PI3K, leading to decreased Akt phosphorylation and thus decreased levels of activation, including decreased IL-2 production and decreased T cell proliferation (62). Engagement of PD-L1 on macrophages to PD-1 promotes IL-10 production, which further promotes immune suppression (93). Currently FDA-approved drugs targeting PD1/PD-L1 for other cancers include the anti-PD1 drug Nivolumab and the anti-PD-L1 drugs pembrolizumab, atezolizumab, and avelumab. No immunotherapeutic drug has been approved to date for glioma.

### TIM-3 and Other Candidates for Adaptive Immune Regulation

As exemplified by exhausted T cells, several additional checkpoint molecules exist besides CTLA-4 and PD-1/PD-L1 that regulate T cell activation and are being assessed as targets for immunotherapy (94). Among these, TIM-3 is expressed by IFNγ-secreting T-helper 1 (Th1) cells, DCs, monocytes, CD8+ T cells, and other lymphocyte subsets (95, 96). TIM-3 is expressed on dysfunctional CD8+ T cells in preclinical models of both solid and hematological malignancies (74, 97). Upregulation of TIM-3 is associated with exhaustion of tumor antigen-specific CD8+ T cells in human melanoma and tumor-induced T cell exhaustion is reversed by administration of anti-TIM-3 antibodies (98, 99). TIM-3 is also expressed on Tregs, with TIM-3+ Tregs identified in solid tumors, such as ovarian, colon, and hepatocellular carcinomas (100). As with other checkpoint molecules, including LAG-3 (73) and TIGIT (75), combination therapies blocking TIM-3 in combination with PD-1 exhibited synergistic effects in preclinical tumor models (74, 101). Kim et al. demonstrated that combination therapy of anti-TIM-3 and anti-PD-1 improved survival in mice with GL261 intra-cranial tumors with optimal outcomes observed using both in combination with stereotactic radiosurgery (76). Several of these checkpoint inhibitors are in clinical trials for GBM (see Supplementary Table 1). Available preclinical data suggest a combined strategy of multiple checkpoint inhibitors with pro-immunogenic interventions, such as stereotactic radiosurgery or oncolytic therapy, may yield optimal outcomes. Much work lies ahead to critically and mechanistically evaluate such combinatorial approaches in clinical trials.

## GBM and the Innate Immune System

### Roles of Innate Immune System in GBM

The innate immune system, comprising CNS-derived microglia, peripherally-derived neutrophils, macrophages, and lymphoid-derived NK cells, has a central role in both glioma and radiation biology (15). In response to CNS inflammation, activated microglia proliferate, secrete cytokines and chemokines, and upregulate cell surface markers such as CD80, CD86, and MHC-II. Microglia also express pattern recognition receptors and cross-present antigens to activate T cells within the CNS (102, 103). Normally absent from the healthy brain, peripherally-derived macrophages are recruited into the GBM microenvironment where they facilitate antigen presentation, immune induction, and removal of cellular debris. Microglia-derived and infiltrating TAMs can comprise up to half the cells in GBM and play a prominent role in tumor growth and invasion (104). Two distinct polarization states of activated macrophages have been frequently described: classically activated “pro-inflammatory” (M1) and alternatively activated “anti-inflammatory” or “chronic inflammatory” (M2) macrophages (105). M1 macrophages serve an important role in phagocytosis of neoplastic cells (106, 107). However, glioma cells can secrete suppressive immune cytokines, such as IL-10 (108), and TGF-β (109), that promote M2 polarization and suppress the M1 phenotype (110). Characterization of TAMs within human GBM has revealed impaired production of pro-inflammatory cytokines, defective antigen-presentation, and poor induction of T cell proliferation (104). Similarly, the GBM microenvironment can also directly render TAMs tolerogenic. GBM cells can induce downregulation of TNF-alpha production, concomitant with induction of anti-inflammatory cytokine IL-10 from microglia through upregulation of STAT 3 and 5 (108).

Another population of peripherally-derived monocytes within GBM are myeloid-derived suppressor cells (MDSCs) that also act to suppress adaptive immunity (111). MDSCs accumulate in GBM, express PD-L1, and impair CD4+ T cell memory function (112). MDSCs lack macrophage-specific markers, such CD68, CD16, and S100A9 (113), and secrete suppressive cytokines, such as TGF-β (114). Though originally described as pleiotropic cells simultaneously expressing both M1 and M2 polarization markers, more recent work has suggested that MDSC are malleable in their polarization phenotype with M1-polarized MDSCs exhibiting tumoricidal properties (115).

Collectively, these studies illustrate the substantial cross-talk between the multiple constituents of the GBM ecosystem in maintaining a milieu conducive to GBM. The therapeutic potential to reprogram TAMs and MDSCs from pro-tumorigenic to tumoricidal polarization states is an area of intense interest. The following sections provide example mechanisms of innate immune system regulation that could be harnessed to anti-tumor effect. To date, radiotherapy has provided a relatively blunt instrument via which to activate the innate immune system. However, limitations include CNS including CNS toxicity and potential for inadvertent activation of pro-tumorigenic sequelae (15). Improved understanding of innate immune mechanisms may provide opportunities to more effectively attack the tumor, whilst protecting against cognitively deleterious effects of radiation.

### Toll-Like Receptor Agonists

Toll-like receptors (TLRs) are pattern recognition receptors (PRRs) expressed by a variety of cell types comprising the innate immune system. The primary function of TLRs is to sense damage and mediate response to pathogens and tumors. TLRs bind to pathogen associated molecular patterns (PAMPs), conserved structures expressed by pathogens, and danger-associated molecular patterns (DAMPs), such as high mobility group box 1 (HMGB1) and fatty acids. TLR 2, 3, 4, and 9 are expressed on human microglia and TAMs (116). DCs also play a prominent role in the development of anti-glioma immunity and anti-tumor response (117). Dead glioma cells release HMGB1 which can activate TLR 2 on DCs, promoting expansion of T cells (118). Preclinical studies with intra-cranial tumors have shown that administration of TLR 3 agonist poly(I:C) attenuated tumor growth in mice (119). Additionally, CpG, in combination with tumor lysate, effectively induced maturation of DCs to control tumor growth (120). Recent work from the Lim laboratory found that that mice treated with poly(I:C) and anti-PD-1 in combination demonstrated increased DC activation, T cell proliferation, and improved tumor control (76). In a phase I clinical study, concomitant administration of DC vaccine, together with adjuvants comprising the TLR7 agonist imiquimod or poly(I:C), appeared safe and increased serum levels of TNF alpha and IL-6 (26). Clinical trials evaluating the safety and efficacy of TLR9 agonist CpG oligodeoxynucleotides demonstrated safety, but no improvement in survival when combined with standard care radiotherapy and TMZ (121–123).

### CD47-SIRP1α Axis

CD47 is a transmembrane immunoglobulin that binds to integrins and serves as a receptor to signal regulatory protein alpha (SIRP1α) and Thrombosponin-1 (TP-1). Expressed on most tumor cells, including GBM (124), CD47 signals “don't eat me” to macrophages. CD47 binding by SIRP1a initiates a signaling cascade that promotes phosphorylation of intracellular ITIMs and activates inhibitory phosphatases SHP-1 and SHP2 (125). These phosphatases dephosphorylate immunoreceptor tyrosine-based activation motifs inhibit pro-phagocytic signals and disrupt cytoskeleton rearrangements necessary for macrophage phagocytosis (125, 126). Antibodies blocking CD47 have been investigated in multiple tumor types to help promote macrophage tumor phagocytosis with efficacy observed in numerous preclinical models, including GBM (124, 127). Clinical trials are underway for both hematologic and solid malignancies (128, 129). Used in combination with radiation, CD47 inhibition has been shown to improve tumor radiosensitivity (130). Anti-CD47 therapy has also been shown to boost antigen presentation (131, 132) and augment cytotoxic CD8+ T cell activity (133). As an adjuvant to radiation therapy, CD47 blockade has the unique advantage of mitigating radiation-induced TSP-1 signaling, which promotes resistance to radiation injury due to decreased inhibition of nitric oxide signaling in normal tissues. As such, whereas most radiation sensitizers increase damage to both tumor and normal tissues alike, the unique biology of CD47 blockade may concurrently enable improved tumor radiosensitivity (via improved phagocytosis) (134), whilst enhancing radioresistance of healthy tissues via increased nitric oxide signaling (130).

### Repolarizing Macrophages

Chemokines, such as colony stimulating factor 1 (CSF-1) and its receptor CSF1R, regulate macrophage homeostasis by regulating proliferation, differentiation, migration, and survival. The intra-tumoral presence of CSF1R-expressing macrophages correlates with poor survival of patients with solid tumors (135). Secretion of CSF-1 by GBM impacts tumor progression through CSF1R signaling. Treatment of GBM with the CSF-1R inhibitor, BLZ945, in transgenic mouse and human xenograft models suppressed tumor growth and improved survival. Although the number of TAMs was not affected, the expression of M2 markers was decreased, consistent with a reduced tumor-supportive phenotype (136). TAMs support tumor progression by blocking anti-tumor immunity and secreting factors to promote angiogenesis (137). TAMs secrete cytokines, such as TGF-β and IL-10, which augment Treg populations while inhibiting effector T cell activity (138). TAMs have been shown to reversibly change their functional phenotype upon exposure to the tumor microenvironment (139). Therefore, strategies that alter the microenvironment to facilitate the repolarization of M2-like TAMs to a M1-tumor-suppressive phenotype are a potential clinical strategy (140).

## Radiation and GBM

### Impact of Radiation on Tumor Immunity

**Figure 1:**
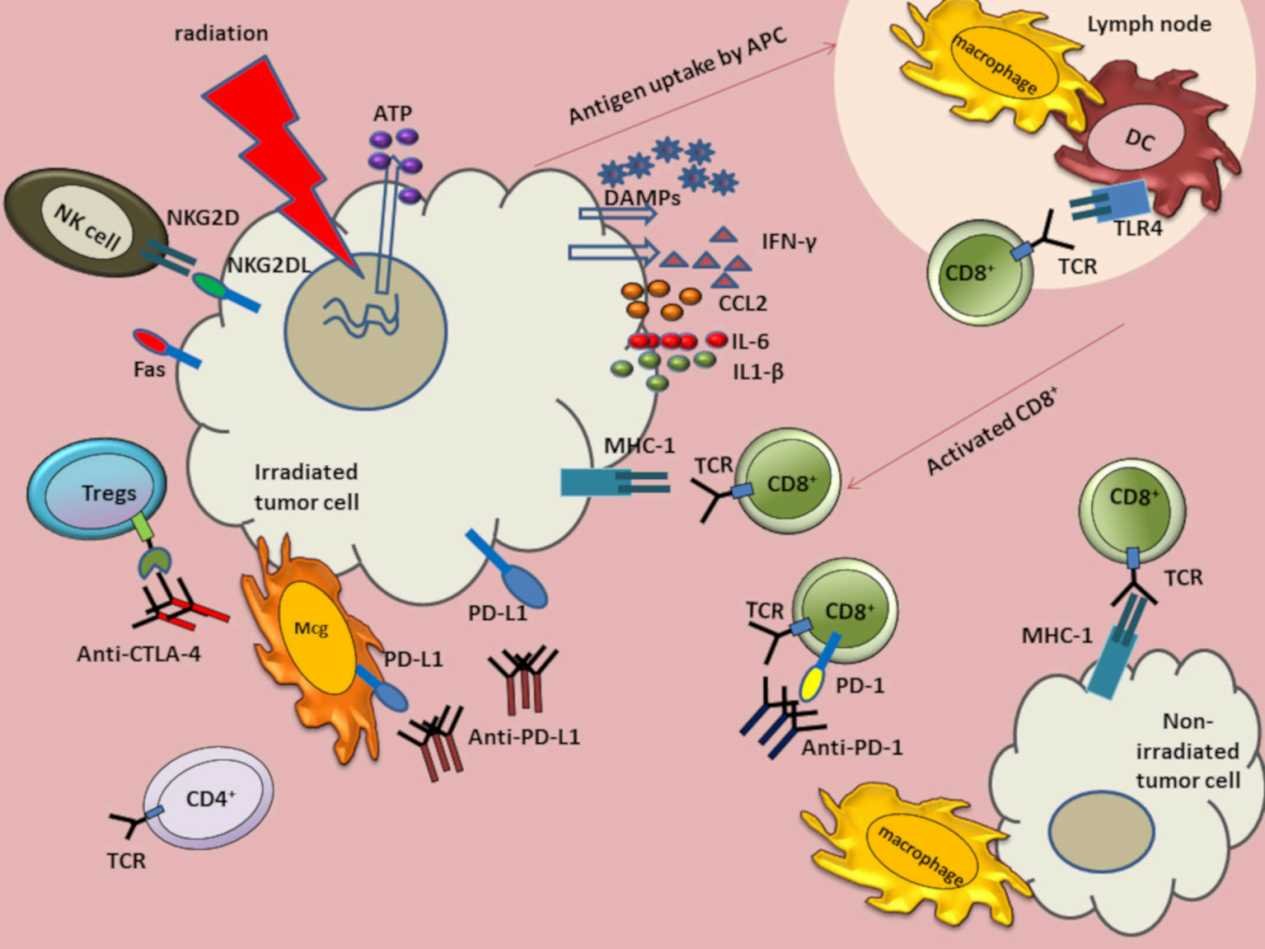
Anti-tumor immune response augmented by the abscopal effect of radiation in combination with immunotherapies. Radiation induces DNA damage and cell death. The dying cells release ATP and DAMPs such as HMGB1 and calreticulin. Although HMGB1 binds TLR4, ATP and calreticulin modulate TLR4 signaling without directly binding to TLR4. Radiation also induces release of tumor antigens to antigen presenting cells (APCs), such as macrophages and dendritic cells (DCs). Antigens are then processed and presented on major histocompatibility complex (MHC) Class I molecules to activate and induce proliferation of CD8+ T cells. The activated cytotoxic CD8+ T cells migrate to tumor sites to induce cell death. Radiation can also induce release of cytokines IL-6 and interferon-gamma (IFN-γ). Radiation also increases tumor cell expression of programmed cell death-1 ligand (PD-L1) and MHC class I molecules. Radiation upregulates immunomodulatory surface proteins, such as Fas and NKG2D ligands on tumor cells. The NKG2D upregulation facilitates NK-mediated tumor cell death. Antibodies, such as α-CTLA-4, α-PD-L1, and α-PD-1 have been used as cancer immunotherapies. When combined with radiation, these antibodies can augment anti-tumor responses in GBM. Anti-CTLA-4 can bind CTLA-4 on Tregs and downregulate suppressive activity. Anti-PDL1 can interact with PD-L1 on tumor cells and on myeloid derived suppressor cells (MDSCs) to curtail suppressive activity induced by MDSCs. Anti-PD-1 antibody can bind to programmed cell death-1 (PD-1) expressed on exhausted T cells.

Radiotherapy is a cornerstone of management for GBM with radiation typically delivered to the enhancing tumor and infiltrative margin via 30 fractions of 2.0 Gy, using IMRT or 3D-conformal therapy. Shorter courses have been considered in elderly patients or as a salvage therapy in recurrent disease. Fractionated radiosurgery has been explored on a trial basis without obviously worse outcomes than standard therapies (141), but has not been adopted in standard management protocols. Radiation acts to ablate dividing cells, induce senescence within non-ablated cells (142). Radiation also stimulates local tumor immunity, promoting anti-tumor immune responses via a host of molecular mechanisms (Figure 1).

MHC class I molecules present intracellular peptide fragments to T cells and are expressed on the surface of all nucleated cells, albeit with reduced expression in tumor and stem cells. MHC class 1 molecules are highly expressed on APCs where they may present phagocytosed peptides from tumors. After activation of APCs, such as DCs, antigens are cross-presented to CD8+ T cells. In the healthy brain parenchyma, microglial cells are the main resident antigen-presenting innate immune cell (143). DCs are also present in the choroid plexus (144). After radiation, the extracellular presence of danger-associated molecular patterns (DAMPs) and cytokines, such as MCP1, contribute to rapid microglial activation (145, 146). We have previously shown that radiation induces a unique polarization state in microglia, which is more closely related to M1 than M2, but distinct from both (147). How the transcriptional responses of human microglia and mouse microglia compare following radiation remains to be determined, though persistent microglial activation has been reported in humans even decades following brain radiation (148). Few lymphocytes are typically found in the healthy brain, despite the role of memory CD4+ memory cells in CNS immunosurveillance (21). Murine brain radiation induces a delayed CNS recruitment of T cells, even in the absence of tumor (149).

NK cells are present in relatively low numbers within the GBM microenvironment, when compared to other tumor types (150). Moreover, these NK cells express relatively low levels of the activating receptor natural killer group 2D (NKG2D) (151). Even within the periphery, patients with GBM demonstrate relatively low numbers of circulating NK cells (152), a number that, like T cells (153), falls further after standard radiation and TMZ (152). NKG2D ligands are potent mediators of both the innate and adaptive immune system (154). Radiation upregulates NKG2D ligands in multiple tumor cell lines, which sensitizes them to NK cell mediated cytotoxicity (110). At present, the impact of radiation on NK cell infiltration into GBM is unclear, though may vary as a function of concomitant TMZ and radiation fractionation schemes.

Although GBM display relatively low levels of surface MHC class I (155), radiation increases MHC class I levels, enhancing cross-presentation of tumor associated antigens in the draining lymph nodes and facilitating recognition of antigenic peptides by CD8+ T cells (156–158). Thus, radiation-induced changes can facilitate activation and proliferation of T cell populations to augment anti-tumor immune response.

Interferon (IFN) levels are robustly elevated following radiation and augment systemic anti-tumor immune response. Of the three distinct types of IFN, types I and II play an important role in sculpting anti-viral and anti-microbial defenses. DNA released from irradiated tumor cells is sensed by stimulator of interferon genes (STING) molecules present on DCs to produce type I IFN. Activation of STING pathway and IFN signaling is required for efficient radiation-induced adaptive immune response (116). IFN-γ, a type II interferon, can upregulate MHC class I and NKG2D expression to increase tumor recognition, inhibit development of Tregs, and increase the induction of cytotoxic T cells (159). Radiation-induced production of IFN-γ by CD8+ T cells augments the immunostimulatory anti-tumor effects of radiation (160).

Interestingly, not all of the pro-inflammatory impacts of radiotherapy necessarily serve to enhance anti-tumoral immunity, illustrating the complexity of regulating immune responses. For example, INF-γ and hypoxia—both of which are induced by radiation—upregulate PD-L1 expression on tumor and tumor-associated immune cells (161, 162). Consistent with this finding, anti-PD-L1 therapy has demonstrated synergistic impacts with radiation to promote anti-tumor immunity (161, 163); results that have been found in metastatic melanoma to be further enhanced by deploying radiation in combination with dual checkpoint blockade (164). Recent data in preclinical models indicate the same may likely hold true in GBM (76).

### Abscopal Effect—Proof of Principle for Radiation-Induced Immunity

Single tumor radiation has occasionally been clinically reported to decrease growth of tumors at distant sites—a previously poorly-understood phenomenon termed the abscopal (ab: “away from;” scopos: “target”) effect (165). In 2004, Demaria et al. used the growth factor Flt3-Ligand to experimentally enhance numbers of antigen presenting cells providing direct evidence that the abscopal effect is immune mediated and tumor-type specific (166). Numerous studies of metastatic cancers have since demonstrated that radiation in combination with checkpoint inhibitors augment the abscopal effect (167–169). Unlike metastatic cancers for which the abscopal effects may be harnessed to attenuate growth of metastatic lesions elsewhere in the body, GBM is typically restricted to a single (occasionally multifocal) lesion within the CNS. Theoretical limitations of a modest neoantigen repertoire, as well as historically regarded modest CNS immune surveillance, could further confound efforts to elicit an abscopal effect for GBM. Nevertheless, the infiltrative nature of GBM, making it refractory to resection, together with known dose-limiting toxicity of brain radiation, increase motivation to harness abscopal biology against infiltrative tumor cells. Multiple studies have reported that systemic immune status may dictate therapeutic efficacy of radiation (170, 171), providing further impetus to optimize radiation by augmenting immune responsiveness.

### Radiation-Induced Cell Death and Immune Activation

Although radiation alone has proven unable to cure glioma, radiation does kill a subset of tumor cells—particularly those that are rapidly dividing. Such cell death facilitates antigen release, as required for adaptive immunity, and stimulates innate immune responses (Figure 1). Radiation induces several types of DNA damage, including simple and complex double stranded breaks (172) with cytotoxic effects (173). Mechanisms of radiation-induced cell death can include necroptosis (174) and p53-dependent apoptosis (175). Radiation-induced mitotic catastrophe may result from radiation, as characterized by aberrant nuclear morphology, multiple nuclei or micronuclei, typically leading to cell death when cells subsequently attempt to divide. However, a small subset of cells may survive with aneuploid or poplyloid karyotypes (176).

Immune activation, as augmented by radiation-induced cell death, facilitates subsequent activation of both the innate and adaptive immune systems against the tumor (177). Immunogenic cell death is mediated by the release of DAMPs directly by tumors or by inflammatory cells present in the microenvironment. Radiation may promote immune activation and immunogenic cell death via at least three mechanisms.

1. **Translocation of Calreticulin (CRT):** CRT is a DAMP that is typically restricted to the endoplasmic reticulum. Translocation of CRT to the cell surface of dying cells stimulates DCs to cross-present antigens to cytotoxic T cells (178).
2. **Extracellular release of HMGB1 and ATP:** Extracellular HMGB1 induces DC activation through TLR-4. TLRs play an essential role in activation of APCs (179) and microglia (180), as well as release of pro-inflammatory signals, including IFN-γ (156, 160). The physical interaction between HMGB1 and TLR4 further prompts optimal cross-presentation of antigens derived from tumor cells by DCs to T cells (181). ATP release from dying cells can also trigger IL-1- β production and priming of CD8+ T cells by activating P2RX7 and PR2Y2 receptors on DCs and macrophages, respectively (182).
3. **Translocation of heat shock proteins:** Cell surface expression of heat shock proteins HSP70 and HSP90 on dying cells induces NK cell activation and promotes cross-presentation of tumor antigens to facilitate DC maturation. Given tumor cell death releases tumor-specific antigens to APCs, including DCs, such cross-presentation of antigens to cytotoxic CD8+ T cells facilitates an anti-tumor T cell response (177, 183).
4. **Upregulated Fas expression:** Garnett et al. have demonstrated radiation increases surface expression of Fas on tumor cells, which augments their destruction by antigen-specific immune effector cells via Fas-dependent mechanisms (184). Binding of Fas, a plasma membrane death receptor protein, to its extracellular ligand, Fas-L, activates caspase 3 and triggers apoptosis. The Fas-FasL axis is integral to maintenance of regulation of immune homeostasis (185, 186) and CD8+ T cell-mediated cytotoxicity (187). CD8+ T cell cytotoxicity is a multi-step process in which the effector cells act to induce cell death by forming cell–cell contacts with potential target cells expressing cell death triggering ligands. Following MHC- antigen recognition, CD8+ T cells lyse target cells via secretion of granzyme and perforin and by the engagement of FasL on T cells with Fas expressed on target cells. Both pathways lead to apoptotic cell death (188).

### Preclinical Data Supporting Combined Radiation and Immunotherapy for GBM

Multiple preclinical studies provide robust proof of principle supporting the combined role of radiation and immunotherapy for GBM (64, 76, 189). In an orthotopic (intracranial) GL261 mouse model, median survival doubled from 27 days with anti-PD1 antibody alone and 28 days in radiation alone, to 53 days when the two modalities were combined. Immunohistochemistry confirmed increased tumor infiltration of cytotoxic CD8+ T cells and decreased regulatory CD4+ T cells in the combination group (64). Similarly, combined radiation and use of an agonist antibody for the co-stimulatory molecule glucocorticoid-induced TNF receptor (GITR) expressed on both regulatory and cytotoxic T cells yielded a cure rate of 24%, compared to 0% for radiation or anti-GITR therapy alone (190).

GL261 is a widely employed mouse GBM line that permits studies in immunocompetent animals (191, 192). As such, many of the seminal studies of immunotherapy with or without radiation have utilized this model. Nevertheless, some have criticized the GL261 model as more highly immunogenic than the immunologically “cold” GBM, thereby potentially over-estimating the clinical potential of immunotherapies for GBM. Numerous genetically engineered models of GBM have been developed, several of which have been well described as “transplantable GEM models” and provide important immunocompetent alternatives to GL261.

As noted in earlier sections, multiple immune checkpoints and other immmunosuppressive strategies are harnessed by GBM to avert immune detection (193). Accordingly, as with preclinical models of metastatic disease, preclinical GBM models have similarly demonstrated improved outcomes with multimodal immune therapy in combination with radiation. Combined use of CTLA-4 blocking antibodies and pro-cytotoxic function CD137 (4-1BB) agonist antibodies with RT yielded 50% survival at 100 days in a GL261 orthotopic model, compared to 20% without RT, and 0% with radiotherapy alone (189). Radiotherapy plus dual checkpoint antibodies against PD-1 and TIM-3 yielded 100% survival of GL261-bearing mice at 100 days, compared to 60% with the best combination of only two of the three treatment modalities (76). Both of these radiation plus dual immunotherapy studies documented elevated CD8+ and CD4+ T cells within the tumor of combined therapy-treated animals (76, 189). Belcaid et al. further performed depletion studies to find that CD4+ but not CD8+ T cells were required for the survival benefit of combined therapy (189).

### Optimizing Radiotherapy for Immune Stimulation

Most clinical trials of immunotherapy, to date, have enrolled patients with recurrent disease following prior standard therapy. As such, patients would have previously undergone radiation and chemotherapy, though would not typically receive further radiation as part of the trial protocol. As such, it is important to note that Belcaid et al. found a trend toward improved outcomes with concurrent, rather than sequential, administration of radiation and immunotherapy (189). Additionally, prior exposure to TMZ attenuates the immune response to checkpoint inhibitors (194).

Current standard therapy for GBM includes chemotherapy and fractionated radiation, frequently also with administration of corticosteroids, which collectively induce lymphopenia and immune suppression (195–198). Importantly, mathematical modeling indicates that even the fractionated radiation to the tumor itself accounts for lymphotoxic doses of radiation to the entire circulating blood pool, even independent of immunosuppressive chemotherapy and steroids (199). As such, stereotactic radiosurgery has been evaluated as an alternative to standard fractionated radiation with a goal of decreasing immunosuppression and increasing tumor ablation and immune activation. Also of note, traumatic brain injury leads to immune suppression via ill-defined mechanisms (200). Whether additional such mechanisms may further impede immune function following brain radiation, independent of lymphodepletion, remains similarly ill-defined. Most GBMs in humans exceed the size limit (~3 cm) considered acceptable for single fraction radiosurgery, though fractionated radiosurgery has been explored with demonstration of feasibility in a preliminary dose-escalation study (141).

With the increasing clinical prevalence and importance of immune-based strategies, attention has focused on how best to harness immune-activating impacts of radiation. The linear quadratic equation is used to determine which fractionated radiation regimens yield equivalent biologically effective doses (201). Importantly, recent data have revealed that too much radiation in a single fraction may inhibit the very immune mechanism one is attempting to activate through radiation-induced immune activation. In an OVA murine melanoma model, 7.5 Gy/fraction yielded best tumor immunity while minimizing numbers of Tregs (202). Radiation doses above 12 Gy were recently found to activate DNA exonuclease Trex1, which decreases DNA from the cytosol and thereby reduces immunogenicity (203). Current efforts to optimize fractionation schemes to optimize RT-mediated immune activation were recently reviewed elsewhere (204). Importantly, optimal parameters appear tumor-dependent. Few studies to date have addressed this question for GBM, though dedicated clinical trials may be needed to elucidate optimal parameters for human patients. A dose escalation study (25–40 Gy) using 5 Gy/fraction with 5 mm margins revealed a maximum tolerated dose of 40 Gy in 8 fractions and an overall survival of 15 months–similar to standard therapy. Further work would be needed to assess relative efficacy of immunotherapies in such novel paradigms compared to that seen with conventional therapy.

## Immunotherapy for Low-Grade Gliomas

The role of immunotherapy for low grade infiltrative gliomas remains poorly characterized. Preclinical efforts in this domain are hampered by the paucity of available animal models. Low-grade gliomas are ultimately fatal due to transformation into high-grade gliomas. Clinical application of immunotherapies for low-grade gliomas are hampered by the lack of biomarkers for efficacy and prolonged periods of relative clinical stability with existing therapies. Low-grade gliomas demonstrate less immunosuppressive phenotypes compared to high-grade gliomas (196, 205–208). This could portend an improved capacity for inducing an immune response, particularly in the context of a more indolent lesion that affords more time to achieve a therapeutic response before the patient would otherwise succumb to disease (209, 210). Conversely, most low-grade gliomas are IDH-mutant and overproduce 2-hydroxyglutarate, which has been found to be immunosuppressive (211). Nevertheless, the specific IDH1 (R132H) mutation itself could serve as a potential vaccine target (212). Preliminary safety trials of vaccines have been performed in pediatric patients with low-grade glioma. A Poly-IC-containing synthetic peptide-based vaccine against the glioma-associated antigens EphA2, IL-13Rα2, and survivin yielded notable immunologic and radiologic responses in a subset of patients (209, 210, 213). Further work is needed to elucidate prospectively which patients and tumor subtypes could benefit from immunotherapy and how favorable responses can be made more consistent.

## Is Radiation and Immunotherapy Relevant to Targeting Glioma Stem Cells?

Cancer stem cells (CSC) have been identified in numerous tumors and play a role in development, invasion, and metastasis. Glioma stem cells (GSC) (82, 214), represent tumor-initiating cells notable for markers of neural stem cell markers, such as CD133 (214) and Nestin (215). Upregulated markers of pluripotent stem cells, including nanog and Oct4, have also been reported (216). GCS demonstrate therapeutic resistance in part through upregulation of DNA damage checkpoint responses and enhanced DNA repair (217). Radiation can induce de-differentiation of GBM cells into a stem cell-like phenotype with increased self-renewal and tumorigenesis capacity in a survivin-dependent manner (218).

GSCs are primarily enriched in the perivascular niche (219). Both microglia and TAMs are found in the perivascular niche and GSC play a prominent role in immunomodulation by recruiting microglia and TAMs. For example, GSCs secrete periostin to recruit TAMs that largely exhibit an M2 phenotype (220). GSCs have also been shown to activate TLR4 on microglia to induce IL-6 secretion (221). Immune therapies against GSCs have included peptide and DC vaccines. Cantini et al. reported in a GL261that vaccination with GLAST, a CNS protein enriched on radial glial cells, promoted tumor immunity without evidence of autoimmunity (222). DC-based vaccines have been explored using tumor lysate or GSC-associated peptides to stimulate *ex vivo* DCs. Administration of loaded DCs in human patients induces prolonged anti-tumor immunity against a potentially broad range of antigens (223). In a GL261-murine model, Pellegatta et al. demonstrated that vaccination using CSC antigens yielded improved anti-tumor effects of DC vaccination when compared with vaccination using regular tumor antigens (224). Similarly, in a rat model, Xu et al. showed that rats vaccinated with GSC-enriched lysates from neurospheres survived longer than rats vaccinated with non-GSC-enriched lysates (225). In recent years, such strategies to target GSCs have been extended to clinical trials.

### A Word of Caution

Immune targeting of GSCs ideally seeks to promote immune responses against antigens uniquely expressed on GSCs, but not healthy tissues. However, care may be needed to ensure that rare endogenous tissue stem cells (neural stem cells or oligodendrocyte progenitor cells) are not inadvertently targeted. Currently, this question is complicated in part by controversy surrounding the presence and identity of adult human endogenous neural stem cells (226). Since GSCs likely reactivate more primitive developmental programs than adult CNS or other tissue progenitor populations, targeting these most primitive markers may help minimize depletion of adult progenitor populations. Since the phenotypes of certain human endogenous progenitor populations remains ill-defined, vigilance for cognitive or other toxicities should be maintained in any therapies potentially inducing auto-immunity against non-mutant endogenous peptides.

## Adjunctive Tools to Promote Tumor Immunity

### DC Vaccines

DCs are one of the most important APCs and have prompted several groups to develop DC-based vaccines for GBM (27, 227–230). DCs have a high capacity to detect maturation signals and process antigens as peptides to generate an efficient and sustained T cell response (231, 232). In an early clinical study of standard chemoradiotherapy followed by GSC-pulsed DC vaccine, 7/11 enrolled patients completed treatment with a median survival of 694 days (233). Currently, it is unclear which factors impact the efficacy of DC vaccination. However, a pre-clinical study by Mitchell et al. showed that DC migration to tumor draining lymphnodes could be enhanced by exogenous administration of the chemokine CCL3 (234). In addition, the authors demonstrated that modulation of CMV-specific DCs with a potent tetanus/diphtheria antigen increased the migratory capacity of DCs and improved the clinical outcomes in patients with GBM (234). A DC vaccine (ICT-107) loaded with six synthetically processed GBM associated peptides (tumor stem cell antigen MAGE-1, her-2, AIM-2, Trp-2, gp100, and IL-13 Rα2) yielded improved progression-free survival and a trend toward improved survival in a randomized, double-blind, placebo-controlled phase II clinical trial for newly diagnosed GBM; however, the study did not meet the primary endpoint of improved overall survival (235). A phase III study was begun, but suspended due to insufficient funding. An initial report demonstrated a median overall survival of 23.1 months in the intention-to-treat population (236). To date, clinical trials have deployed DC therapies following completion of standard chemoradiation therapy. Whether or not modifications to standard therapy could further augment DC-mediated responses remains to be investigated.

### Targeted Immunotherapy

#### Epidermal Growth Factor Receptor Variant III Vaccines

Epidermal growth factor receptor (EGFR) variant III (vIII) is expressed in 20–30% of GBM (237). EGFRvIII is absent in normal tissues and selective activation of PI3K/Akt pathway contributes to GBM resistance to radiotherapy (238). Work by Heimberger et al. demonstrated that immunization of DCs mixed with a tumor-specific peptide of EGFRvIII, PEP-3 conjugated to the immune adjuvant keyhole limpet hemocyanin (KLH), resulted in long-term survival of mice with intracranial melanomas (239). The vaccine Rindopepimut, which targets EGFRvIII, has shown efficacy in phase I/II clinical trials, but demonstrated no survival benefit in a phase III trial (see Supplementary Table 1) (240, 241).

#### Survivin

Survivin, a regulator of both mitosis and programmed cell death (242), is a tumor associated antigen, making it an attractive candidate for targeted cancer therapy and immunotherapy (242–244). Normal glial cells do not express survivin, whereas survivin is highly expressed in GBM and is associated with poorer prognosis (245). Epitopes of survivin are immunogenic and are presented by MHC Class I complexes. Anti-survivin antibodies have been identified in patients with GBM (246). In an effort to identify a survivin peptide mimic that could elicit a potent T cell response, Ciesielski et al. created SVN53-67/M57, a peptide vaccine derived from survivin. SVN53-67/M57 produced cytotoxic T cell-mediated killing of human glioma cells *in vitro* and, in combination with GM-CSF, was able to control tumor burden in mice bearing GL-261 glioma tumors (247). A phase II trial of SVN53-67/M57-KLH (SurVaxM) and TMZ is currently recruiting patients with malignant glioma and the therapy has shown to be well tolerated and generates anti-survivin antibody and survivin specific CD8+ T cells (248).

#### Oncolytic Viruses

While this review focuses particularly on the facilitating role of radiation in checkpoint blockade, oncolytic viruses may serve a similar role by means of immune activation in GBM (249). Although oncolytic viruses are selected or engineered for their propensity to replicate or selectively kill tumors cells, complete viral-induced lysis of all tumor cells is not observed with the relatively attenuated viral constructs clinically deployed, to date. Instead, the lysis of a subset of tumor cells may serve to promote both anti-viral and anti-tumor immune responses (250). The combination of measles virus-expressing carcinoembryonic antigen with radiation has been shown to improve tumor control (251). Similarly, the combination of radiation with oncolytic DNA viruses, such as herpes-simplex virus-1 and conditionally replicating adenovirus, has demonstrated longer survival and improved outcomes in pre-clinical GBM models (252–254). Despite some case reports of remarkable responses, clinical trials of oncolytic therapies for GBM have proven disappointing, to date, with only marginal therapeutic efficacy reported (249) (ClinicalTrials.gov, Unique Identifier: NCT01280552)^1^. These findings have prompted ongoing efforts to both better predict which patient populations may respond favorably and how responses may be further augmented.

## Clinical Translation: Challenges and Practical Considerations

**Figure 2:**
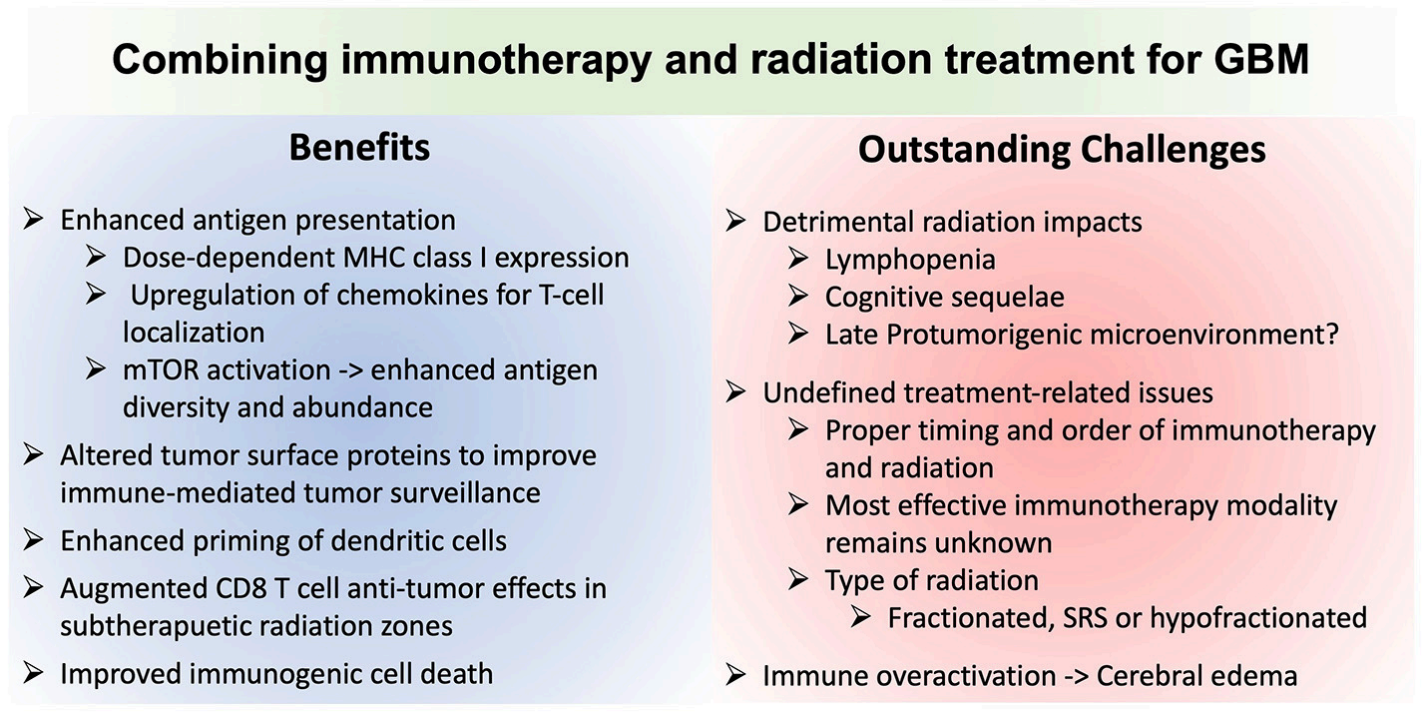
Comparison of the advantages and potential challenges of combining immunotherapy and radiation for glioblastoma treatment. MHCI, Major histocompatibility complex class I molecule; mTOR, mechanistic target of rapamycin; SRS, stereotactic radiosurgery.

Translating immunotherapy for GBM has proven challenging. Optimally harnessing radiation to augment the efficacy of immunotherapy is a promising avenue, but is not without its own unique challenges (Figure 2). While many patients seeking clinical trials have recurrent disease, prior radiation may preclude further radiation due to risk of toxicity and may impact immune responses in ways that are difficult to predict. While TMZ may attenuate bone marrow immune responses, TMZ-induced mutations may provide important neoantigens to catalyze immune recognition of the tumor.

Tumor heterogeneity remains a challenge, both within and between patients. Furthermore, human immune responses are complex and will likely require molecular and genetic subtyping to identify potential subclasses and individual “responders” or “partial responders.” For example, the phase II ICT-107 autologous DC vaccine trial suggested clinical responses only in subjects who were HLA-A2 positive (a phase III trial was suspended for financial reasons). Several immunotherapy clinical trials are ongoing for GBM, which is routinely treated with radiation, including DC vaccines, EGFRvIII vaccines, and checkpoint inhibitors, among others. However, few studies, to date, have specifically focused on optimizing synergy between radiation and immunotherapy.

GBM is transcriptionally subclassified into proneural, neural, classical, and mesenchymal based on genomic profiling (255). However, single cell transcriptome data suggest variable representations of each transcriptional cell type within each tumor, challenging selective targeting of the tumor phenotype. Moreover, radiation has been shown to induce a mesenchymal phenotype, notable for its poorest prognosis; likely due in part to radiation-induced upregulation of treatment-resistant stem-like properties (256). Data from other tumor types suggest that cytokines from local tissue in response to immunotherapies may offer an important source of more reliable biomarkers, including biomarkers of therapeutic responsiveness (257). If also true in glioma, this may create impetus to identify technologies for *in vivo* evaluation of such biomarkers locally within the tumor microenvironment in response to therapy—an avenue our group is currently exploring.

The paucity of prompt biological feedback regarding efficacy remains a challenge. While systemic immune cell populations can be serially accessed to monitor leukocyte numbers and phenotypes, these data are at best an indirect and imperfect indicator of therapeutic efficacy within the tumor. Imaging criteria to interpret immunotherapy responses, despite interpretations challenges of radiation- and immunotherapy-induced pseudoprogression, have been drafted (iRANO). The lack of definitive imaging biomarkers of responsiveness is underscored by the need to follow the trajectory of imaging changes over months to interpret findings (258).

Finally, it is increasingly appreciated that standard management strategies aside from radiation likely inhibit the efficacy of immunotherapy, including immuosuppressive corticosteroids and systemic chemotherapy. Corticosteroids, such as dexamethasone, are used to control vasogenic edema due to infiltrative tumor, surgery, and radiotherapy (259). Pre-clinical models and retrospective data from clinical studies indicate that dexamethasone treatment attenuates the efficacy of radiotherapy, presumably by impeding normal radiation-induced immune responses (260). While TMZ is the cornerstone of the standard STUPP regimen for GBM, experimental data demonstrate that systemic chemotherapy impedes the anti-tumor effects of anti-PD-1, despite the potential for local chemotherapy to augment immunotherapeutic responses (194). These studies highlight practical challenges of optimizing the therapeutic impacts of immunotherapy. Until methods can better predict responses or evaluate therapeutic impact in real time, forgoing the established standard of care (TMZ) to theoretically augment an unproven experimental therapy may prove challenging. Our group recently initiated a clinical trial providing anti-PD-1 in biopsy-proven GBM prior to definitive surgical resection and subsequent chemo/radiation. Insights from early histological analysis of tissue from patients treated with anti-PD-1 may help identify biomarkers and selection criteria for future single and combination immunotherapy trials (ClinicalTrials.gov, Unique Identifier: NCT03197506). As increasing evidence emerges about untoward chronic impacts of radiation on the CNS microenvironment for tumor aggressiveness, could future paradigms replace standard fractionated radiation with combination immunotherapy and hypofractionated SRS applied to just a portion of the tumor? Alternatively, perhaps residual tumor cells after chemo/radiation may be best eliminated with combined immunotherapy and senolytic therapy? Finally, strategies are needed to optimally titrate the immune response to avert potentially severe or fatal toxicities. These may vary in a tumor- and patient-specific manner based on biomarkers of susceptibility and responses that have yet to be identified. We posit that dedicated efforts to understand the human biology of CNS radiation and therapeutic responses may reveal opportunities to optimize safety and efficacy of combined radiation and immunotherapy for glioma.

## Conclusions

The dramatic anti-tumor clinical responses observed in certain tumors treated with anti-CTLA-4 and anti-PD-1 antibodies have ushered in a new era for effective cancer therapies. Radiation modulates the tumor microenvironment and offers a potential immune adjuvant to enhance the anti-tumor response in combination with immunotherapies. Preclinical models of GBM illustrate potent opportunities to harness combination immunotherapy with brain radiation. However, several questions remain unanswered regarding the optimal paradigms of combination immunotherapy, timing in relation to radiation, and the potential to mitigate adverse impacts of currently standard treatments, such as fractionated radiotherapy-induced lymphopenia and chemotherapy- and corticosteroid-induced immunosuppression. Preclinical evidence suggests robust opportunities to add optimized strategies of immunotherapy into standard-of-care for GBM. Much work lies ahead to improve translational paradigms that could increase mechanistic insights gleaned from each treated patient and enable iterative improvements in protocols within the life-times of individual patients.

## Author Contributions

KR, LC, and TB conceived the aim of the review, performed the literature search, and wrote the manuscript with additional intellectual contributions from IP, AJ and AW. KR drafted Figure 1, LC drafted the Supplementary Table 1 and Figure 2.

### Conflict of Interest Statement

The authors declare that the research was conducted in the absence of any commercial or financial relationships that could be construed as a potential conflict of interest. The handling editor declared a shared affiliation, though no other collaboration, with the authors at the time of review.

## Footnotes

^1^Pembrolizumab and Standard Therapy in Treating Patients With Glioblastoma. Available online at: https://clinicaltrials.gov/ct2/show/NCT03197506.
